# TGF-β-Induced CD8^+^CD103^+^ Regulatory T Cells Show Potent Therapeutic Effect on Chronic Graft-versus-Host Disease Lupus by Suppressing B Cells

**DOI:** 10.3389/fimmu.2018.00035

**Published:** 2018-01-30

**Authors:** Haowen Zhong, Ya Liu, Zhenjian Xu, Peifeng Liang, Hui Yang, Xiao Zhang, Jun Zhao, Junzhen Chen, Sha Fu, Ying Tang, Jun Lv, Julie Wang, Nancy Olsen, Anping Xu, Song Guo Zheng

**Affiliations:** ^1^Department of Nephrology, Sun Yat-sen Memorial Hospital of Sun Yat-sen University, Guangzhou, China; ^2^Department of Clinical Immunology, The Third Affiliate Hospital of Sun Yat-sen University, Guangzhou, China; ^3^Department of Nephrology, The Affiliated Hospital of Xuzhou Medical University, Xuzhou, China; ^4^Guangdong Provincial Key Laboratory of Malignant Tumor Epigenetics and Gene Regulation, Sun Yat-sen Memorial Hospital of Sun Yat-sen University, Guangzhou, China; ^5^Division of Rheumatology, Milton S. Hershey Medical Center, Penn State University, Hershey, PA, United States

**Keywords:** TGF-β1, CD8^+^CD103^+^ iTreg, lupus nephritis, immunosuppression, B cell responses

## Abstract

Lupus nephritis is one of most severe complications of systemic erythematosus lupus and current approaches are not curative for lupus nephritis. Although CD4^+^Foxp3^+^ regulatory T cells (Treg) are crucial for prevention of autoimmunity, the therapeutic effect of these cells on lupus nephritis is not satisfactory. We previously reported that CD8^+^CD103^+^ Treg induced *ex vivo* with TGF-β1 and IL-2 (CD8^+^CD103^+^ iTreg), regardless of Foxp3 expression, displayed potent immunosuppressive effect on Th cell response and had therapeutic effect on Th cell-mediated colitis. Here, we tested whether CD8^+^CD103^+^ iTreg can ameliorate lupus nephritis and determined potential molecular mechanisms. Adoptive transfer of CD8^+^CD103^+^ iTreg but not control cells to chronic graft-versus-host disease with a typical lupus syndrome showed decreased levels of autoantibodies and proteinuria, reduced renal pathological lesions, lowered renal deposition of IgG/C3, and improved survival. CD8^+^CD103^+^ iTreg cells suppressed not only T helper cells but also B cell responses directly that may involve in both TGF-β and IL-10 signals. Using RNA-seq, we demonstrated CD8^+^CD103^+^ iTreg have its own unique expression profiles of transcription factors. Thus, current study has identified and extended the target cells of CD8^+^CD103^+^ iTreg and provided a possible application of this new iTreg subset on lupus nephritis and other autoimmune diseases.

## Introduction

Systemic lupus erythematosus (SLE) is a serious autoimmune disease with incompletely understood pathogenesis. A major immune feature of SLE is the hypersecretion of autoantibodies by dysfunctionally autoreactive B cells (1). Lupus nephritis, a foremost cause of morbidity and mortality, is present in up to 60% SLE patients (2), and features immune complex deposition in the glomerulus, consequently causing tissue damage and proteinuria. Therapeutic approaches available include immunosuppressive drugs, biologicals, and corticosteroids (3). Among these, B cell depletion using rituximab has been used for the treatment of SLE and many autoimmune and chronic inflammatory diseases (4, 5), but its role is restricted for incapable of depleting long-lived plasma cells (6). All of these treatment options are not a permanent cure which would be one that ideally reverses immune imbalance.

Regulatory T cells (Treg) are a subset of T cells that maintain self-tolerance by suppressing autoreactive lymphocytes, mainly consisting of natural occurring Treg cells (nTreg) and induced Treg cells (iTreg) (7, 8). We and others have widely reported that both nTreg and iTreg have immunosuppressive properties and exert therapeutic effects on autoimmune diseases (9–14). However, CD4^+^Foxp3^+^ nTreg showed instability in inflammatory conditions and their therapeutic effects on the established autoimmune diseases were sometimes unsatisfactory (15, 16). It has been shown that some CD4^+^Foxp3^+^ nTreg had converted to Th17 cells after encountering IL-6 and other inflammatory cytokines (15, 16), although CD4^+^Foxp3^+^ iTreg might be more stable in the inflammation environment (15, 17–19).

Although Foxp3 is essential for the development and function of CD4^+^ Treg cells, it is not a case for CD8^+^ iTreg cells. We previously reported that CD8^+^CD103^+^ iTreg induced *ex vivo* with TGF-β and IL-2 potently suppressed Th cell response and Th1/Th17-mediated colitis, regardless of Foxp3 expression (20). CD8^+^Foxp3^+^CD103^+^ iTreg and CD8^+^Foxp3^−^CD103^+^ iTreg shared similar immunosuppressive capability in suppress Th cell response, while CD8^+^CD103^−^ T cells showed no inhibition ability. These studies imply that CD8^+^CD103^+^ iTreg may have some advantages in treating inflammatory diseases since their role is not dependent upon Foxp3 expression. As CD4^+^Foxp3^+^ nTreg cells had a minimal therapeutic effect on lupus nephritis (11), we were interested in exploring whether CD8^+^CD103^+^ iTreg have therapeutic effect on SLE/lupus nephritis.

In the current article, we show that infusion of CD8^+^CD103^+^ iTreg to lupus mice displayed a potent therapeutic effect on lupus nephritis. CD8^+^CD103^+^ iTreg reduced autoantibody titers and proteinuria, decreased renal pathological lesions, as well as diminished IgG and C3 deposition in renal glomerulus. Further observation demonstrated that the therapeutic effect is greatly dependent on the direct suppression of B cell responses which involve both TGF-β and IL-10 signals. RNAseq technology further identified that CD8^+^CD103^+^ iTreg have a unique expression profile of transcription factors that distinguishes them from CD4^+^ Treg cells.

## Results

### Infusion of CD8^+^CD103^+^ iTreg Cells Significantly Ameliorates Lupus Nephritis

**Figure 1:**
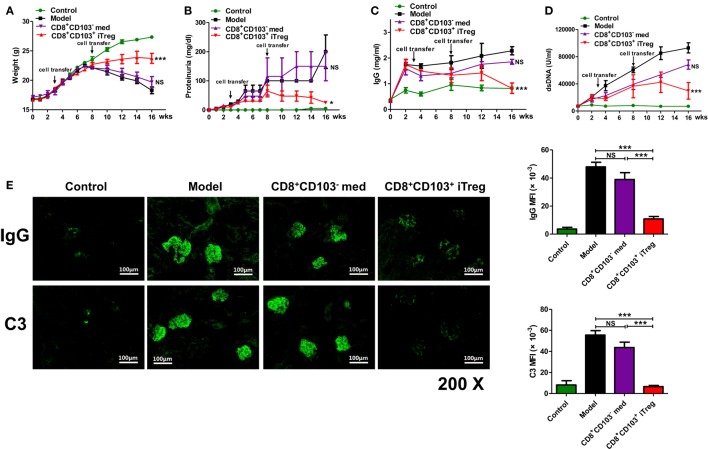
CD8^+^CD103^+^ iTregs show potent therapeutic effect on chronic graft-versus-host disease (cGVHD) lupus nephritis mice. CD8^+^CD103^−^ med, CD8^+^CD103^+^ iTregs induced from DBA/2 mice were adoptively transferred to cGVHD lupus nephritis mice at 3 and 8 weeks. There were four mice in each group. **(A–D)** CD8^+^CD103^+^ iTreg cells significantly reversed the decrease in weight, and the increase in proteinuria in lupus nephritis mice after 8 weeks, and also prevented the continuous rise in dsDNA Ab and total IgG titers. The data indicate the mean ± SEM of four individuals (NS means no significance, *P < 0.05, ***P < 0.001, CD8^+^CD103^−^ med or CD8^+^CD103^+^ iTreg versus model). **(E)** CD8^+^CD103^+^ iTregs reduced IgG or C3 immune deposition in the glomeruli, IgG or C3 mean fluorescence intensity are significantly lower in CD8^+^CD103^+^ iTreg group. The data indicate the mean ± SEM of four independent experiments (NS means no significance, ***P < 0.001).

To determine the therapeutic effect of CD8^+^CD103^+^ iTregs on lupus nephritis mice, we have used chronic graft-versus-host disease (cGVHD) mice as established lupus nephritis model (21, 22). Naive CD8^+^ cells isolated from DBA/2 mouse were stimulated with anti-CD3/CD28 coating beads and IL-2 in the absence (CD8 Med) and presence (CD8 iTreg) of TGF-β for 3 days, and then CD8^+^CD103^−^ cells were sorted from CD8 Med as CD8 control cells (CD8 Med), CD8^+^CD103^+^ cells were sorted from iTreg cells as CD8^+^CD103^+^ iTreg cells as previously described (20). Adoptive transfer of DBA2 spleen cells to DBA2xC57BL/6 F1 mouse will develop a typical lupus syndrome characterized by increased levels of IgG autoantibody on the first week and proteinuria on the eighth week after cell transfer, providing an ideal model to study SLE/lupus nephritis. CD8^+^CD103^+^ iTreg or CD8^+^CD103^−^ were transferred into chronic GVHD mice at 3 and 8 weeks after DBA2 cell transfer. Infusion of CD8^+^CD103^+^ iTreg cells significantly reversed the decrease of weight, the increase of proteinuria in mice after 8 weeks, whereas CD8^+^CD103^−^ control cells did not show these effects (Figures 1A,B).

We also determined effects on serum dsDNA Ab and total IgG titers. CD8^+^CD103^+^ iTreg prevented the continuous rise in total IgG and dsDNA Ab titers after cell transfer. The levels of dsDNA Ab and total IgG were significantly lower in cGVHD mice that received CD8^+^CD103^+^ iTreg than in cGVHD, although infusion of CD8^+^CD103^−^ cells was also slightly decreased the levels during 8–14 weeks (Figures 1C,D).

All mice were sacrificed for pathological examination of kidneys at 16 weeks post DBA/2 cell transfer. Immunofluorescence staining in kidney revealed that the IgG or C3 immune deposition in the glomeruli of cGVHD mice that received CD8^+^CD103^+^ iTreg was reduced compared to the untreated cGVHD model mice or cGVHD mice that received CD8^+^CD103^−^ control cells. IgG or C3 mean fluorescence intensity (MFI) of glomeruli was significantly lower in CD8^+^CD103^+^ iTreg group versus control groups (Figure 1E).

**Figure 2:**
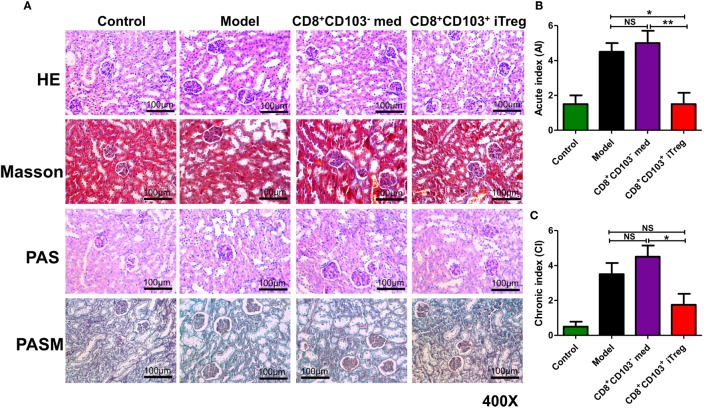
CD8^+^CD103^+^ iTregs reduced renal pathological lesions in lupus nephritis mice. CD8^+^CD103^−^ med, CD8^+^CD103^+^ iTregs were adoptively transferred to chronic graft-versus-host disease lupus nephritis mice at 3 and 8 weeks. There were four mice in each group in one experiment. **(A)** CD8^+^CD103^+^ iTregs alleviated the renal pathologic lesion. **(B,C)** Lupus nephritis mice with CD8^+^CD103^+^ iTreg treatment had lower disease activity and chronicity indices. The data indicate the mean ± SEM of four independent experiments (NS means no significance, *P < 0.05, **P < 0.01).

Using HE, Masson, periodic acid-Schiff (PAS), or periodic acid-silver metheramine (PASM) staining on kidney paraffin sections, we observed that renal pathologic lesions were much less in cGVHD mice treated with CD8^+^CD103^+^ iTreg compared to control groups (Figure 2A). CD8^+^CD103^+^ iTreg treatment also resulted in a significant lower degree of disease activity and chronicity indices (Figures 2B,C), while cGVHD model group and CD8^+^CD103^−^ cell treatment group mice exhibited typical pathological damage of lupus nephritis, as shown by disease activity and chronicity index scores (Figures 2A–C).

### CD8^+^CD103^+^ iTreg Cells Suppress B Cell Responses *Ex Vivo*

**Figure 3:**
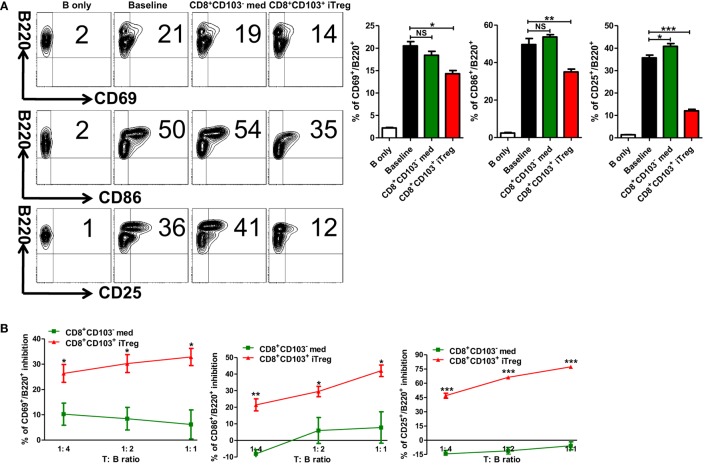
CD8^+^CD103^+^ iTregs directly suppress B cell responses *ex vivo*. **(A)** B cells were isolated from C57BL/6, stimulated with (baseline) or without lipopolysaccharide (B only) in the presence or absence of CD8^+^CD103^−^ med or CD8^+^CD103^+^ iTregs (T:B = 1:2). The expression of B cell early activation (CD69, CD86) and later activation (CD25) markers was detected after 48 h of culture by flow cytometry. Typical FACS plots and summary data are shown. The data indicate the mean ± SEM of three independent experiments. (NS means no significance, *P < 0.05, **P < 0.01, ***P < 0.001.) **(B)** Then, B cells cocultured with CD8^+^CD103^−^ med or CD8^+^CD103^+^ iTreg in the same way (ratio of T to B cells was 1:4 to 1:1). The inhibition percentages are given. The data indicate the mean ± SEM of three independent experiments (*P < 0.05, **P < 0.01, ***P < 0.001, CD8^+^CD103^+^ iTreg versus CD8^+^CD103^−^ med at the same ratio).

A previous study has demonstrated that CD8^+^CD103^+^ iTreg mainly suppress Th cells (20). Given that B cells play an important role in the pathogenesis and development of lupus and lupus nephritis (23, 24), we also sought to determine whether CD8^+^CD103^+^ iTregs can directly suppress B cells. The first experiment was carried out using an *ex vivo* assay. CD8^+^CD103^+^ iTreg or control cells and B cells were cocultured, and B cell activation and proliferation, including the ability of B cells to produce antibodies in the presence of lipopolysaccharide (LPS) were analyzed at different time points. Compared with the CD8^+^CD103^−^ control cells, CD8^+^CD103^+^ iTregs markedly suppressed the expression of CD25, CD69, CD86 on B cells (Figure 3A), indicating that CD8^+^CD103^+^ iTreg cells may directly suppress B cell activation. We further studied the gradient effects of this suppressive capacity at the ratio of 1:1 to 1:4 (T: B) and which shows a dose-dependent effect (Figure 3B). CD8^+^CD103^+^ iTregs also suppressed the expression of CD138 while control cells slightly reduced the expression with no significance (Figure S1 in Supplementary Material).

**Figure 4:**
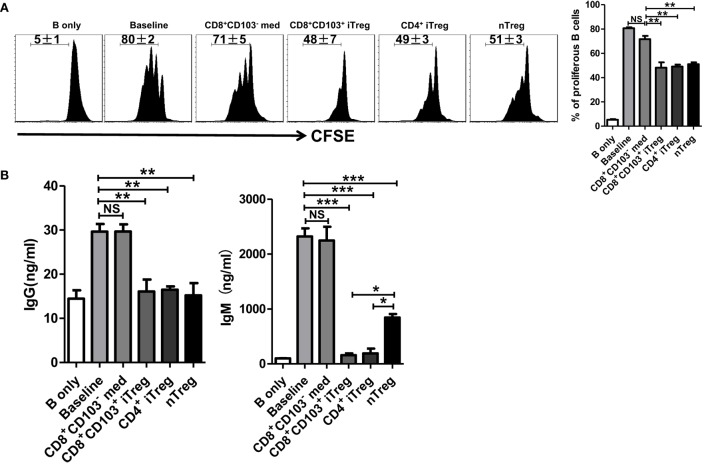
CD8^+^CD103^+^ iTregs directly suppress B cell proliferation and the ability to secrete antibody *ex vivo*. **(A)** Fresh B cells labeled with CFSE were cultured in 96-well plates with CD8^+^CD103^−^ med, CD8^+^CD103^+^ iTreg, CD4^+^ iTreg, or nTreg cells (the ratio of T cells: B cells was 1:2) in the presence of lipopolysaccharide. B cell proliferation was determined by the CFSE dilution rates after 3 days of culture. Typical FACS histogram and summary data were shown. **(B)** The supernatants were collected from T cells and B cells co-culture systems after 3-day culture, and the IgG and IgM secretion was detected by ELISA. The data indicate the mean ± SEM of three independent experiments (NS means no significance, *P < 0.05, **P < 0.01, ***P < 0.001).

We next determined whether CD8^+^CD103^+^ iTreg cells also suppress B cell proliferation. B cells have been labeled with CFSE that enables quantitation of cell proliferation. As shown in Figure 4A, CD8^+^CD103^+^ iTregs but not CD8^+^CD103^−^ control cells markedly suppressed B cell proliferation and the inhibitory efficiency was comparable to that of CD4^+^ iTreg or nTreg subsets.

One of mostly important features of B cells is the ability to produce antibodies. We therefore also analyzed the ability of CD8^+^CD103^+^ iTregs or CD8^+^CD103^−^ control cells to regulate antibody production by B cells. As expected, CD8^+^CD103^+^ iTreg significantly suppressed IgG and dramatically inhibited IgM production when these cells were cocultured with LPS-stimulated B cells. Conversely, CD8^+^CD103^−^ control cells did not display any suppressive effect on antibody production (Figure 4B). Thus, CD8^+^CD103^+^ iTreg cells at least demonstrate their suppression of B cells similar to their partners of CD4^+^ Treg cells (25–28).

### Cell Contact and TGF-β/IL-10 Signals Are All Needed for Suppressive Effect of CD8^+^CD103^+^ iTreg Cells on B Cell Responses

**Figure 5:**
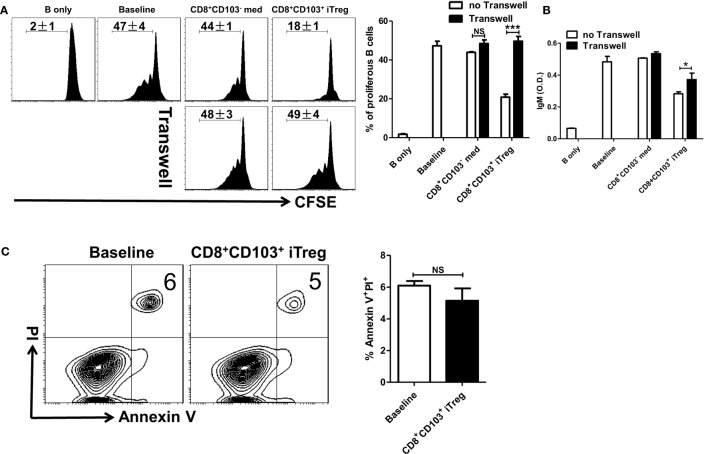
CD8^+^CD103^+^ iTregs suppress B cell responses by a cell contact dependent mechanism. **(A,B)** Fresh B cells labeled with CFSE were cultured in 24-well plates with CD8^+^CD103^−^ med or CD8^+^CD103^+^ iTreg (the ratio of T cells: B cells was 1:2) in the presence of lipopolysaccharide (LPS), with or without Transwell (0.4 μM). The proliferation was analyzed by the flow cytometry. The supernatants were collected from T cells and B cells co-culture systems after 3-day culture, and the IgM secretion was measured by an ELISA. Typical FACS histogram and summary data are shown. **(C)** CD8^+^CD103^+^ iTreg cells were cocultured with B cells in the presence of LPS. After 16 h of culture, the apoptosis percentage of B220^+^ cells was detected by flow cytometry using the Annexin V/PI kit. Representative FACS plots were gated on B220^+^ cells and summarized data were shown. The data indicate the mean ± SEM of three independent experiments (NS means no significance, ***P < 0.001).

We further explored underlying mechanisms whereby CD8^+^CD103^+^ iTreg cells suppress B cell responses. B cells and CD8^+^CD103^+^ iTreg cells were cocultured through either cell contact or Transwell system that enables isolation of both cell populations while allowing soluble molecules secreted from Treg cells to permeate the B cell compartment. The proliferative levels and antibody secretion of LPS-stimulated B cells in the presence of either CD8^+^ iTreg or control cells were measured to evaluate the inhibitory ability of CD8^+^ cells. We observed that CD8^+^CD103^+^ iTregs suppressed the B cell proliferation and antibody secretion when cell–cell is present in the culture, but this ability to suppress B cells proliferation disappeared when CD8^+^ Treg cells were isolated from B responder cells. The ability to suppress the antibody secretion of B cells was also weaken under Transwell situation. CD8^+^ control cells did not suppress B cell functions, regardless of the presence or absence of cell contact (Figures 5A,B).

We also extended our study to determine whether CD8^+^CD103^+^ iTregs suppress B cell responses *ex vivo* through TGF-β or/and IL-10 signals. As shown in Figure S2 in Supplementary Material, TGF-β or/and IL-10 signals were indeed needed for their suppressive effects on B cell responses *ex vivo*. It is likely that CD8^+^CD103^+^ iTreg cells act target cells *via* their secretion of active TGF-β and TGF-β binding on membrane-bound (cell surface) receptors.

### CD8^+^CD103^+^ iTreg Cells Suppress B Cell Responses That Is Independent upon Cytotoxicity

Given that nTreg directly suppress B cell responses by cytotoxic mechanisms (26, 28), largely by secreting the cytotoxic molecules granzyme A, granzyme B, and perforin; and CD4^+^ iTreg directly suppress B cell responses through a non-cytotoxic mechanism involving TGF-β signaling (25), we explored the possibility whether cell killing is involved in the inhibitory effect of CD8^+^CD103^+^ iTregs on B cell responses.

CD8^+^CD103^+^ iTreg cells were cocultured with B cells in the presence of LPS. After 16 h of coculture, apoptosis percentage of B220^+^ cells was detected by flow cytometry. We found that CD8^+^CD103^+^ iTregs did not promote apoptosis of B cells in the coculture system (Figure 5C). We also conducted coculture measurements at different time points, 48 and 72 h, and no significant B cell apoptosis change was observed (data not shown). Then we observed that cytotoxic molecules including granzyme A, granzyme B, and perforin were no expressed in CD8^+^CD103^+^ iTreg cells (Figure S3 in Supplementary Material), which strengths the conclusion that CD8^+^CD103^+^ iTreg suppress B cell responses by non-cytotoxicity way.

### CD8^+^CD103^+^ iTreg Suppress B Cell Responses *In Vivo*

**Figure 6:**
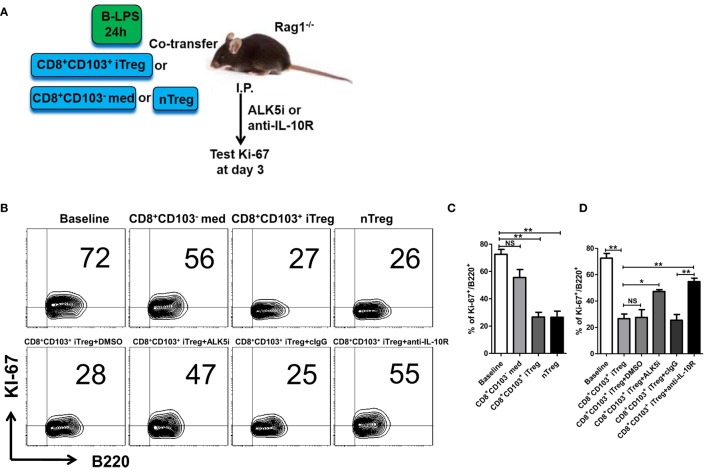
CD8^+^CD103^+^ iTregs directly suppress allogeneic B cell responses *in vivo*. **(A)** Fresh B cells were stimulated with lipopolysaccharide (LPS) for 24 h and then cotransferred with CD8^+^CD103^−^ med, CD8^+^CD103^+^ iTreg, or nTreg cells (T: B = 2:1, B cells 8 million per mouse) into Rag1^−/−^ mice, in some groups, ALK5i (1 mg/kg), anti-IL-10R Ab (1 mg/kg), DMSO (for ALK5i control), or control IgG were i.p. injected at days 0 and 2. The mice were sacrificed at day 3, and splenic B cells were harvested and prepared to test Ki-67 expression on B cells by flow cytometry. **(B,C)** Co-transfer of CD8^+^CD103^+^ iTregs or nTregs significantly reversed the high expression of Ki-67 in B cells *in vivo*. **(B,D)** Blockade of TβRI or IL-10R greatly abolished the suppressive effect of CD8^+^CD103^+^ iTregs on B cells proliferation in *in vivo*. There were three mice in each group. The data indicate mean ± SEM of three independent experiments (NS means no significance, *P < 0.05, **P < 0.01).

The results generated from *in vitro* experiments do not necessarily reflect the consequences *in vivo*. To determine the role and mechanisms of CD8^+^ iTreg against B cell responses, we carried out the *in vivo* experiments as established previously to address this possibility (25). B cells pretreated with LPS were cotransferred with CD8^+^CD103^−^ med, CD8^+^CD103^+^ iTreg, or nTreg cells (2:1 ratio) into Rag1^−/−^ mice; in some groups, TGF-β receptor I inhibitor (TβRI inhibitor, ALK5i), anti-IL-10R Ab, control IgG, or DMSO (for Alk5i control) were injected i.p. 3 days after cell transfer (Figure 6A). Ki-67 expression in LPS-stimulated B cells is markedly increased in this model. Co-transfer of CD8^+^CD103^+^ iTregs or nTregs significantly reversed the high expression of Ki-67 in B cells *in vivo*, while co-transfer of CD8^+^CD103^−^ med cells only slightly reduced Ki-67 expression, without statistical significance (Figures 6B,C).

We observed that suppression of CD8^+^CD103^+^ iTregs on B cell responses *in vivo* was largely dependent upon TGF-β or/and IL-10 signals, because blockade of TβRI or IL-10R greatly abolished the suppressive effect (Figure 6D). Of interest, the IL-10 signal seemed to have a more important role in the suppression of CD8^+^CD103^+^ iTregs on B cells *in vivo*, whereas the suppression of CD4^+^ iTregs on B cell responses was mostly dependent on the TGF-β signal and partly dependent on the IL-10 signal (25).

**Figure 7:**
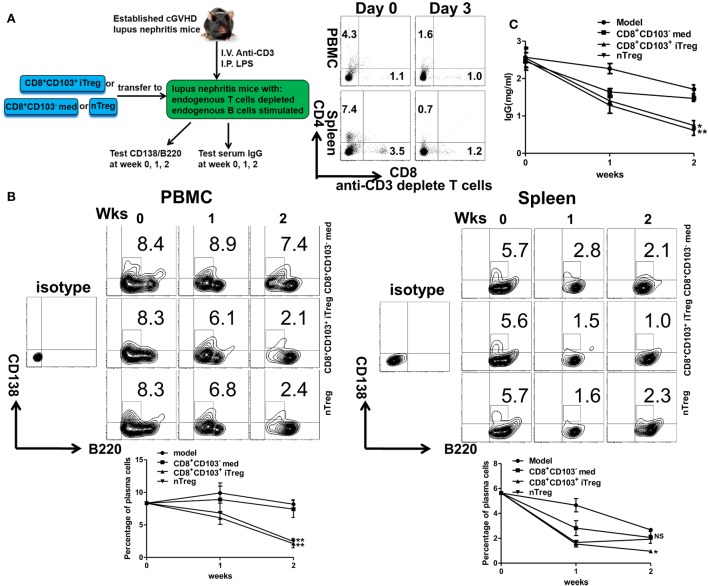
CD8^+^CD103^+^ iTregs directly suppress autoreactive B cells in chronic graft-versus-host disease (cGVHD) lupus nephritis mice. **(A)** CD8^+^CD103^−^ med, CD8^+^CD103^+^ iTreg, or nTreg cells were adoptively transferred to B6D2F1 cGVHD lupus nephritis mice with high titers of anti-dsDNA and proteinuria. These lupus nephritis mice were previously depleted of endogenous T cells (300 mg anti-mouse CD3 Ab, i.v.) and had activation of B cells [10 μg lipopolysaccharide (LPS) per mouse, i.p.] 3 days before the cell transfer. There were three mice in each group. On day 3, there were almost no CD4^+^ or CD8^+^ T cells in cGVHD mice with anti-CD3 depletion. **(B)** The percentage of CD138/B220 (CD138 is plasma cell marker) in PBMC or spleen was measured in different lupus nephritis mice groups at the 0-, 1-, and 2-week time points after cell transfer. **(C)** The serum total IgG titer at 0-, 1-, and 2-week time points after cell transfer were detected by ELISA. The data indicate the mean ± SEM of three independent experiments. (NS means no significance, *P < 0.05, **P < 0.01, CD8^+^CD103^−^ med, or CD8^+^CD103^+^ iTreg versus model.)

Further evidence that CD8^+^CD103^+^ iTregs suppress B cell response *in vivo* was provided using the lupus model. CD8^+^CD103^−^ med, CD8^+^CD103^+^ iTreg, or nTreg cells were adoptively transferred to cGVHD lupus mice with proteinuria of greater than100 mg/dl, in which endogenous T cells had been previously deleted and endogenous B cells had been previously stimulated (Figure 7A). The results showed that the percentages of peripheral blood CD138^+^ plasma cells were significantly decreased in CD8^+^CD103^+^ iTreg or nTreg group compared with the CD8^+^CD103^−^ group 2 weeks post cell transfer. CD8^+^CD103^+^ iTregs also significantly reduced the percentages of splenic CD138^+^ plasma cells, but nTregs did not show such suppressive ability at similar time points (Figure 7B). Nonetheless, both CD8^+^CD103^+^ iTregs and nTregs similarly suppressed IgG production in cGVHD lupus mice 2 weeks after cell transfer (Figure 7C). These results suggest that CD8^+^CD103^+^ iTregs suppress not only allogenic B cells but also autoreactive B cell responses *in vivo*.

Although previous studies have shown that B cells express TGF-β receptor I (TβRI) (29) and IL-10 receptor α (IL-10Rα) (30), it is not clear whether the expression of this two receptors were changed during the cGVHD lupus-like disease model establishing process. Real-time PCR experiments were carried out and we observed that the expression of TβRI and IL-10Rα on B cells was not changed during the model processing (Figure S4 in Supplementary Material). Thus, it is reasonable that CD8^+^CD103^+^ iTreg suppress B cell responses *via* TGF-β and IL-10 signals.

### RNA-seq Identifies That CD8^+^CD103^+^ iTreg Is a Unique Treg Population

**Figure 8:**
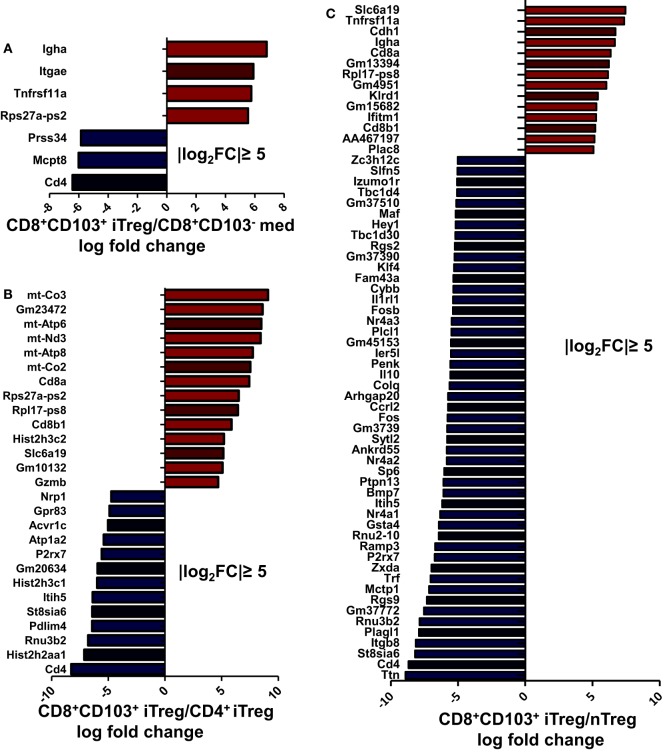
Each regulatory T cells (Treg) subsets has its own gene expression profile. CD8^+^CD103^−^ med, CD8^+^CD103^+^ iTreg, CD4^+^ iTreg, and nTreg cells were prepared, RNA was extracted, and DNA libraries were constructed. RNAseq was conducted on an Illumina HiSeq platform following the manufacturer’s instructions. **(A–C)** Significantly altered genes fold change (FC) plotted for specific two cell subsets are shown, with the filter of |log_2_FC| ≥ 5.

To determine and compare possible differences between CD8^+^CD103^+^ Treg cells and other Treg subsets or non-Treg cells, we carried out RNA-seq analysis for differentially expressed genes in CD8^+^CD103^−^ med, CD8^+^CD103^+^ iTreg, CD4^+^ iTreg, and nTreg subsets. A total 48,440 genes were detected using fastx-toolkit v0.0.14 and cutadapt v1.7.1. There were 323 significantly differentially expressed genes in genes expression heatmap for the four different cell subsets (Figure S5A in Supplementary Material). Pairwise genes expression comparison histograms of CD8^+^CD103^−^ med versus CD8^+^CD103^+^ iTreg, CD8^+^CD103^+^ iTreg versus CD4^+^ iTreg, CD8^+^CD103^+^ iTreg versus nTreg are also shown (Figures 8A–C), using the filter of |log_2_FoldChange| ≥ 5 [fold change (FC)]. Each cell subset has its own specific gene profile that may be used as a tool to distinguish one population from another.

Compared with CD8^+^CD103^−^ non-Treg cells, CD8^+^CD103^+^ iTreg cells have higher expression of Igha, Itgae, and Tnfrsf11a. It is reasonable to expect that CD103 coded by gene Itgae would be highly expressed on the CD103^+^ cell population. CD103 expression has been shown to be an essential molecule for CD8^+^ iTregs to suppress Th cell responses (20) and may be centrally involved in the suppressive effect on B cell responses. CD265, also known as receptor activator of NF-κB (RANK), coded by gene Tnfrsf11a, may be a specific marker for CD8^+^CD103^+^ iTregs which may also play an important role in immunosuppressive function of CD8^+^CD103^+^ iTregs (Figure S5B in Supplementary Material). Previous studies have demonstrated that CD265 is involved in T cell/dendritic cell interactions and tolerance induction (31, 32).

After applying the filter of |log_2_FC| ≥ 5, a total of 27 genes showed differential expression between CD8^+^CD103^+^ iTreg and CD4^+^ iTreg, of which 14 were upregulated in CD8^+^CD103^+^ iTreg and 13 were upregulated in CD4^+^ iTreg (Figure 8B). With the same filter, a total of 63 genes showed differential expression between CD8^+^CD103^+^ iTreg and nTreg, of which 13 were upregulated in CD8^+^CD103^+^ iTreg and 50 were upregulated in nTreg (Figure 8C).

## Discussion

Thymus-derived CD4^+^CD25^+^ T regulatory cells (nTreg) maintain immune tolerance and possess immunosuppressive capacity (33). Although nTreg cells have an ideal role in preventing autoimmune and inflammatory diseases (9–11), the therapeutic effect of these cells on established diseases including lupus nehpritis is fairly unsatisfactory since they are unstable and dysfunctional in inflammation conditions (15, 16, 34–38), CD4^+^ Treg cells induced *ex vivo* provide a new Treg population and have displayed advantages in the inflammation conditions (12–15, 39–41), showed the therapeutic effect on established autoimmune diseases (12, 18), but others have reported that Foxp3 CPG is highly methylated in CD4^+^ iTreg cells, raising a concern about whether CD4^+^ iTregs can sustain long-term therapeutic effects on autoimmune diseases (42).

We recently reported that CD8^+^CD103^+^ Treg cells induced *ex vivo* have a similar functional characteristic compared to CD4^+^ nTregs and iTregs. Interestingly, these Treg cells suppress autoimmune diseases independent of Foxp3 expression (20). They need both TGF-β and IL-10 to suppress immune responses distinguishing them from Tr1 and Th3 cells (43, 44). New evidence provided from current study demonstrates that CD8^+^CD103^+^iTreg suppress lupus B cells also *via* TGF-β and IL-10 signals since these lupus B cells indeed maintain the expression of TGF-β and IL-10 receptors.

CD103, the αEβ7 integrin, is a receptor for the epithelial cell-specific ligand E-cadherin that was first reported expressed on CD8^+^ cytolytic T lymphocytes. CD103 plays a crucial role in responding to allogeneic epithelial cells and in affecting allograft survival (45). In a rat liver transplantation model, the levels of CD8^+^ T cells with upregulated CD103 expression were associated with long-time survival of allograft recipients (46). In another mouse renal transplantation model, it was found that the development of CD8^+^CD103^+^ cells depends on TGF-β signaling and facilitates their migration to renal allograft (47). In eye-derived tolerance, CD103 is necessary for CD8^+^ T cells regulatory mechanisms (48). Thus, CD103 expression may help distinguish the CD8^+^ cell Treg population from non-Treg cell population and may also participate in maintaining immune balance or in immunosuppression in diverse diseases (49, 50).

Besides CD103, we found some potential specific markers for CD8^+^CD103^+^ iTreg according to RNA-seq data. CD265 might be a specific marker, as previous studies have demonstrated that CD265 is involved in T cell/dendritic cell interactions and tolerance induction (31, 32). However, more data are needed to address this possibility including the development of CD265 KO mouse. Although CD226 also seems to be another specific marker for CD8^+^CD103^+^ iTreg, the previous study also showed that CD226 is a costimulatory molecule and plays an important role in activation and effector functions of Th1 cells (51), making CD226 less likely to be a specific marker for CD8^+^CD103^+^ iTreg.

In human SLE, the spontaneous activity of antibody-forming B cells is increased in the peripheral blood (52). In established autoimmune disease models, dysfunction of B cells also exists (53). As the most important part of humoral immunity, B cells play a crucial role in occurrence and development of autoimmune diseases mostly through the abnormal secretion of autoAbs, presentation of autoantigens, abnormal secretion of inflammatory cytokines, modulation of Ag processing, and generation of heterotopic germinal centers (6). A recent study revealed that B cells are major source of pro-inflammatory IL-6 and a key driver of lupus nephritis (54). In addition to its role in humoral immunity, IL-6 also sabotages the functional activity of Treg cells (15, 16, 55).

Hence, the depletion of B cells or suppression of B cell function represents a new therapeutic strategy in patients with SLE and other autoimmune diseases. Anti-IL-6 antibody has been approved for the treatment of patients with rheumatoid arthritis and may extend to patients with SLE (56) or other conditions. In addition, application of Treg cells may have a potential therapeutic effect, since several Treg subsets can suppress B cells. Although nTreg cells kill B cells, that may not happen in patients with SLE (26, 28) and CD4^+^ iTreg cells suppress B cells *via* a non-cytotoxic mechanism that may provide an advantage in clinical applications (25).

To overcome the concern that Foxp3 is unstable in inflammatory conditions, we have studied the functional characteristics of CD8^+^CD103^+^ iTreg cells in a lupus nephritis model. These cells not only strongly suppressed LPS-stimulated B cell responses, and reduced the levels of serum IgG and IgM secreted by B cells in lupus, but also prevented renal pathologic damage. Unlike nTreg cells, these cells suppress B cells independent of cell killing. RNA-seq technique identified that this is a unique Treg cell population, distinguishing them from nTreg and CD4^+^ Treg cells. Given their role does not need Foxp3 expression, it is likely that they are more stable and functional in the presence of inflammation. We had also conducted experiment where we transferred CD8^+^CD103^+^ iTregs or nTregs population into RAG1^−/−^ mice and look their stability in different time points. The results turned out that the CD103 expression in CD8^+^CD103^+^ iTregs is more stable than the Foxp3 expression in nTreg (Figure S6 in Supplementary Material).

Although other CD8^+^ Treg cell subsets, such as CD8^+^CD28^low/−^ or CD8^+^CD122^+^ cells, also suppress immune response, CD8^+^CD103^+^ Treg subset might be different from them. First, they did not completely share the phenotypic similarities, for example, CD8^+^CD103^+^ Treg cells are both CD122^+^ and CD122^−^, these cells also express somehow CD28 (20). Second, both CD122^+^ and CD28^low/−^ cells can be produced naturally or developmentally. Last, CD8^+^CD28^low/−^ cells suppress immune responses *via* IL-10 but not TGF-β although CD8^+^CD122^+^ cells requires both IL-10 and TGF-β (57). More studies including gene profiles are needed to fully distinguish these cell subsets.

Taken together, we now reveal that CD8^+^CD103^+^ iTreg induced *ex vivo* significantly control the appearance and development of nephritis in lupus-like diseases; therefore, use of CD8^+^CD103^+^ iTregs may have a potential promise for the treatment of lupus nephritis and other autoimmune and inflammatory diseases.

## Materials and Methods

### Mice

Female C57BL/6 (B6) mice were purchased from Guangdong Medical Laboratory Animal Center (Guangzhou, CHN), 6- to 8-week-old female DBA/2 mice and (C57BL/6 × DBA/2) B6D2DF1 mice were purchased from Vital River (Beijing, CHN) and Jackson Lab (USA). RAG1^−/−^ mice (B6.129S7-Rag1tm1Mom/JNju) were purchased from Nanjing Biomedical Research Institute of Nanjing University (Nanjing, China) and Jackson Lab (USA). This study was carried out in accordance with the recommendations of Sun Yat-sen University for the Use and Care of Animals (Approval No. IACUC- DB-16-0909) and Milton S. Hershey Medical Center (IACUC NO. 46887). The protocol was approved by the Sun Yat-sen University and Penn State University.

### Flow Cytometry

The following fluorescent mouse Abs from Biolegend (San Diego, CA, USA) were used for flow cytometry analysis: CD3, CD4, CD8, CD103, CD25, CD62L, CD69, CD86, CD138, Foxp3, and Ki-67; from eBioscience (San Diego, CA, USA): B220, granzyme B, perforin; from Santa Cruz (Dallas, TX, USA): granzyme A. Cell subsets were stained with mAbs and isotype control as indicated above and analyzed on BD LSRFortossa™ flow cytometer (BD Biosciences, San Diego, CA, USA) using FACSDiva Software (BD Biosciences). For intracellular staining, such as Foxp3, Granzyme A, GranzymeB, Perforin, and Ki-67, cells were first stained with surface marker, and further fixed and permeabilized for intracellular staining using Fix and Perm (eBioscience). Final plmiceot/histogram figures were prepared using FlowJo Software (Tree Star, Ashland, OR, USA).

### The Generation of CD4^+^ iTreg, CD8^+^CD103^−^ Med, CD8^+^CD103^+^ iTreg, and nTreg

CD4^+^ naive T cells (CD4^+^CD25^−^CD62L^+^CD44^low^) were isolated from splenic cells of C57BL/6 mice using a naive CD4^+^ T cell isolation kit (Miltenyi Biotec, Auburn, GER) (Purity around 95%). CD8^+^ naive T cells (CD8^+^CD25^−^CD62L^+^CD44^low^) were isolated from splenic cells of C57BL/6 or DBA/2 mice, by first staining with Biotin-conjugated (Biolegend): anti- B220, CD4a, CD11b, CD11c, CD25, CD49b, Ter-119, CD44, and then with Biotin beads (Miltenyi Biotec), using AutoMACSpro (Miltenyi Biotec) to negatively select CD8^+^ naive T cells (purity around 95%). Cells were cultured in 48-well plates and stimulated with anti-CD3/CD28-coated beads (one bead per five cells; Life Technologies, Carlsbad, CA, USA) in the presence of IL-2 (50 U/ml; R&D Systems, San Diego, CA, USA) with (CD4^+^/CD8^+^ iTreg) or without (CD8med) TGF-β1 (2 ng/ml; R&D Systems) for 3 days. After 3 days, CD4^+^/CD8^+^ iTreg and CD8med cells were harvested and the beads were removed, then cells were positively selected for CD103 (CD103^+^ around 90%) with Biotin-CD103 (Biolegend) and Biotin beads (Miltenyi Biotec) using AutoMACSpro (Miltenyi Biotec) (purity of CD8^+^CD103^+^ more than 95%); the same method was used to negatively select CD103 in CD8med (purity of CD8^+^CD103^−^ more than 97%). nTreg cells were sorted by FACS using FACSAria™ (BD Biosciences) (purity of CD4^+^CD25^+^ more than 98%), expanded with anti-CD3/CD28-coated beads (one bead per three cells; Invitrogen) and IL-2 (300 U/ml; R&D Systems) for 3 days. A total of 300 U/ml IL-2 was renewed at day 2. After cultures, cells were harvested and beads removed with DynaMagTM (Life Technologies). RPMI 1640 medium supplemented with 100 U/ml penicillin, 100 mg/ml streptomycin, 10 mM HEPES (Gibco, Carlsbad, CA, USA), and 10% heat-inactivated FBS (ExCell Bio, SHH, CHN) was used for all cultures. Foxp3 expression was determined by flow cytometry.

### The Selection of B Cells

B cells were positive selected from spleen cells of C57BL/6 mice with Biotin-B220 (Biolegend) and Biotin Beads (Miltenyi Biotec) using AutoMACSpro (Miltenyi Biotec). The purity of B cells was more than 99%. For determination of B cell proliferation, B cells were labeled with CFSE (Biolegend) before being cocultured with Tregs.

### cGVHD Lupus Nephritis Model and Treg Adoptive Transfer

To establish the cGVHD lupus nephritis model (22, 58), single-cell suspensions of splenic cells from DBA/2 donors were prepared by gently grinding the spleens in a 40 μm filter (BD Falcon) with RPMI 1640. Then erythrocytes were removed with Red Blood Cell Lysing Buffer (Sigma-Aldrich), suspensions were washed twice in PBS and centrifuged for 5 min at 300 *g*, and then cells were resuspended in PBS. The B6D2F1 recipients were injected intravenously in 0 week with 80 × 10^6^ of these viable cells in 0.3 ml PBS. Proteinuria was determined with Semi-quantitative Albustix paper (Gaoerbao, Guangzhou, China). The levels of serum IgG, dsDNA were determined by ELISA every 2 weeks. The mice that had proteinuria were selected to use as the lupus nephritis model. In weeks 3 and 8, 3 × 10^6^ CD8^+^CD103^+^ iTreg or CD8^+^CD103^−^ med cells in 0.3 ml PBS were, respectively, transferred into cGVHD group. Control and model group mice received the same volume of PBS. There were 3–4 mice for each group in one experiment and experiments were repeated with similar results at least 3–4 times. We measured body weight and proteinuria every 2 weeks, the level of dsDNA, IgG in sera were also measured every 2 weeks.

### Pathology and Immunofluorescence

At week 16, all mice were sacrificed for kidney pathology. The kidney tissues were processed for light and immunofluorescence microscopy. The light-microscopic slides were stained with hematoxylin–eosin, Masson, PAS or PASM, and used to calculate the activity and chronicity indices (59, 60) of different groups. Immunofluorescence slides were stained with rabbit anti-mouse IgG (Abcam, Cambs, UK) or rabbit anti-mouse C3 (Santa Cruz), and then stained with goat anti-rabbit IgG (Abcam), observed with fluorescence microscope (Axio Vert A1, ZEISS, Germany), and the MFI of glomerulus in different groups was calculated using ImageJ software (National Institutes of Health, USA).

### *In Vitro* Suppression Assays

To examine the suppressive effect of Treg on B cell *in vitro*, B cells were stimulated with or without LPS (*Escherichia coli* 0111, 5 μg/ml, B4, Sigma-Aldrich, St. Louis, MO, USA) in the presence or absence of graded numbers of CD8^+^CD103^−^ med or CD8^+^CD103^+^ iTreg. The ratio of T cells to B cells ranged from 1:4 to 1:1. In other experiments, B cells were also cocultured with CD8^+^CD103^−^ med, CD8^+^CD103^+^ iTreg, CD4^+^ iTreg, or nTreg cells (the ratio of T: B was 1:2). For determination of the activation and differentiation of B cells, after 2 day coculture, CD69/CD86 (early activation index), CD25 (later activation index), and CD138 (differentiation index) were determined by flow cytometry. For determining the proliferation of B cells, fresh B cells were labeled with CFSE before coculture, and proliferation levels were judged by the intensity of CFSE dilution with flow cytometry after 3 days of coculture.

To determine the mode of suppressive action of the Treg, 0.4 μm Transwell plates (Corning, NY, USA) were used to separate CD8^+^CD103^−^ med/CD8^+^CD103^+^ iTreg and B cells from direct contact during the coculture.

### Apoptosis Assays

CD8^+^CD103^+^ iTregs were cocultured with B cells in the presence or absence of LPS (5 μg/ml, Sigma) for 16, 48, or 72 h. Then cells were collected and stained with Annexin V and propidium iodide (PI) using an Annexin V apoptosis detection kit (Sungene Biotech, Tianjin, China) following the manufacturer’s specifications. Both Annexin V and PI expression were measured by LSRFortossa™ flow cytometer (BD Biosciences), gated on B220^+^ cells.

### Autoantibody Detection

To compare the IgG, IgM Abs production secreted by B cells *in vitro*, the supernatants were collected from abovementioned systems after 3 days of culture. For the *in vivo* autoantibody detection, mice were bled at the indicated time points, and sera were collected. IgG, IgM, and anti-dsDNA were, respectively, measured by IgG ELISA kit (eBioscience), IgM ELISA kit (eBioscience), and dsDNA ELISA kit (Alpha Diagnostic, San Antonio, TX, USA). All samples were performed with triplicate. Sera samples were diluted 1/100 for anti-dsDNA and 1/25,000 for measuring IgG.

### Real-time PCR

Total RNA was extracted from B cells isolated from cGVHD lupus mice or normal control mice using the TRIzol reagent (Invitrogen) according to the manufacturer’s instructions. Reverse transcription (RT) of total RNA was carried out with PrimeScript™ RT reagent Kit (TaKaRa). cDNA Amplification was performed using a Roche LightCycler 480 Sequence Detection System (Roche) with Ssofast EvaGreen supermix (Bio-RAD). Primer sequence were as follows: GAPDH, 5′-GGTTGTCTCC TGCGACTTCA-3′ and 5′-TGGTCCAGGGTTTCTTACTCC-3′; TβRI,5′-CTATGCTGGTCCAGTCTTCG-3′and 5′-TGGTGAATGACAGTGCGGTTATGG-3′;IL-10Rα, 5′-AAGCAATGGACGGCATCATCTATGG-3′and 5′-AACTCGGAGATC CTTGAAGACTTGTTC-3′.

### *In Vivo* Suppression Assays

B cells stimulated with LPS (5 μg/ml) for 24 h were then washed with PBS to remove LPS. These B cells were then cotransferred with CD8^+^CD103^−^ med, CD8^+^CD103^+^ iTreg, or nTreg cells (the ratio of T:B was 1:2, 8 million B cells per mouse) into B6 Rag1^−/−^ mice (6 weeks age). In addition, TβRI inhibitor (ALK5) (1 mg/kg, Sigma-Aldrich), anti-IL-10R (1 mg/kg, Biolegend), or DMSO (control for ALK5i) or cIgG (control for anti-IL-10R) were given to some groups mice by i.p. injection at days 0 and 2. The mice were sacrificed as indicated time points and splenic B cells were harvested and used to measure Ki-67 expression in cells gated on B220^+^ by flow cytometry. Then we carried out another *in vivo* suppression assay. The established cGVHD lupus nephritis mice with evident lupus nephritis were depleted of endogenous CD3^+^ T cells (26) with a single-dose 300 mg anti-mouse CD3 Ab (ExCell Bio, Shanghai, CHN), or with the isotype control, and endogenous B cells were stimulated with a single 10 μg dose of LPS (Sigma-Aldrich). Three days later, CD8^+^CD103^−^ med, CD8^+^CD103^+^ iTregs, or nTregs were adoptively transferred to the mice with anti-CD3 Ab treatment. The percentages of plasma cells (CD138^+^) were detected and sera were collected 0, 1, and 2 weeks after cell transfer for IgG measurement.

### Library Construction and Sequencing, Data Analysis

CD8^+^CD103^−^ med, CD8^+^CD103^+^ iTreg, CD4^+^ iTreg (the Foxp3 expression in CD4^+^CD25^+^ iTreg more than 90%), and nTreg cells were prepared as described, and RNA was extracted using MiniBEST Universal RNA Extraction kit (TaKaRa, Japan). cDNA library was constructed using TruSeq Stranded Total RNA Sample Preparation Kit with Ribo-Zero Gold (Illumina, San Diego, CA, USA). The products were sequenced on an Illumina HiSeq platform using a pair-end 150bp mode, following the manufacturer’s instructions. Raw data were cleaned using fastx-toolkit v0.0.14^1^ and cutadapt v1.7.1.^2^ The clean read-pairs were then aligned to the human reference genome (UCSC hg 19) by Tophat v2.1.1,^3^ read counts were calculated using HTSeq v0.6.1,^4^ and differential expression analysis was performed using DESeq.^5^ The RNA-seq data were submitted to NCBI SRA database, the accession number for RNA-seq is PRJNA419054 (SRP125726).

### Statistical Analysis

Data were expressed as mean ± SEM unless otherwise indicated. Data were analyzed using the unpaired *t* tests for comparison between two groups or ANOVA for comparison among multiple groups as appropriate in SPSS. Comparison between two groups in multiple groups used Bonferroni. Differences were considered statistically significant when *p* < 0.05.

## Ethics Statement

This study was carried out in accordance with the recommendations of Sun Yat-sen University for the Use and Care of Animals (Approval No. IACUC- DB-16-0909) and Milton S. Hershey Medical Center (IACUC No. 46887). The protocol was approved by the Sun Yat-sen University and Penn State University.

## Author Contributions

AX and SZ designed the research topic and charge correspondence; HZ wrote the manuscript; HZ, YL, and ZX designed and carried out all the experiments; PL, HY, XZ, and JZ helped carrying out experiments; JC, SF, YT, and JL analyzed experimental results and analyzed data and developed analysis tools; JW provide assistance and guidance on experiments; NO provide manuscript modification.

## Conflict of Interest Statement

The authors declare that the research was conducted in the absence of any commercial or financial relationships that could be construed as a potential conflict of interest.

## Footnotes

^1^http://hannonlab.cshl.edu/fastx_toolkit/.

^2^https://pypi.python.org/pypi/cutadapt/.

^3^http://ccb.jhu.edu/software/tophat/index.shtml.

^4^https://pypi.python.org/pypi/HTSeq.

^5^http://www.bioconductor.org/packages/release/bioc/html/DESeq.html.
