# Supplementary material for: TGF-β-Induced CD8+CD103+ Regulatory T Cells Show Potent Therapeutic Effect on Chronic Graft-versus-Host Disease Lupus by Suppressing B Cells
Source: Front Immunol. 2018 Jan 30;9:35. doi: 10.3389/fimmu.2018.00035 (PMC5797539; doi:10.3389/fimmu.2018.00035)
Supplement: Supplementary file 1 [file Data_Sheet_1.doc]

**Figure S1**


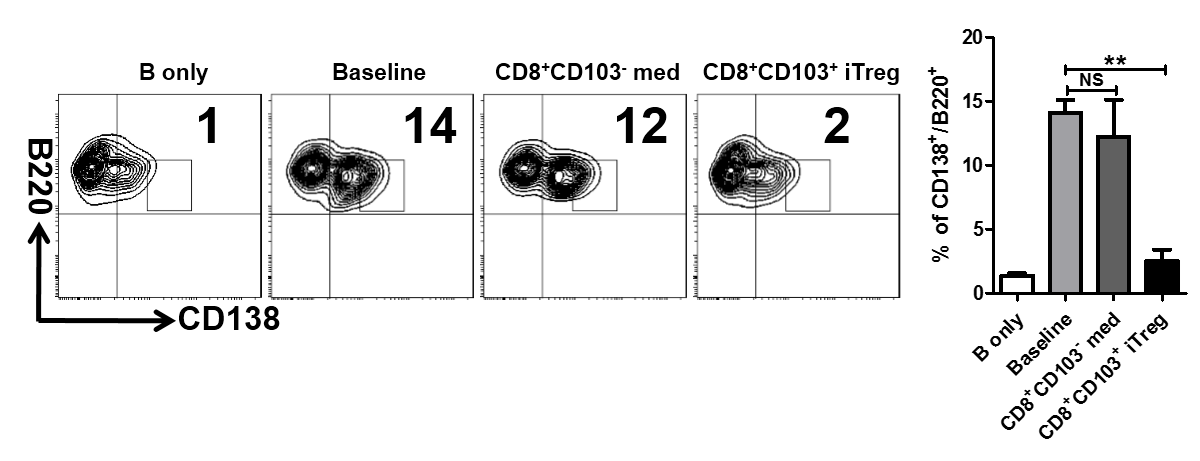


**Figure S1 | CD8^+^CD103^+^ iTreg greatly inhibited the differentation of B cells to plasma cells *ex vivo*.** B cells were isolated from C57BL/6, stimulated with (baseline) or without LPS (B only) in the presence or absence of CD8**^+^**CD103**^-^** med or CD8**^+^**CD103**^+^** iTreg (T: B= 1:2). The percentage of plamsa cells in B cells (B220^low^CD138^high^ cell subset) was detected after 48 h of culture by ﬂow cytometry. Typical FACS plots and summary data were shown. The data indicate the mean ± SEM of three independent experiments. (NS means no significance, ***P<*0.01)

**Figure S2**

**
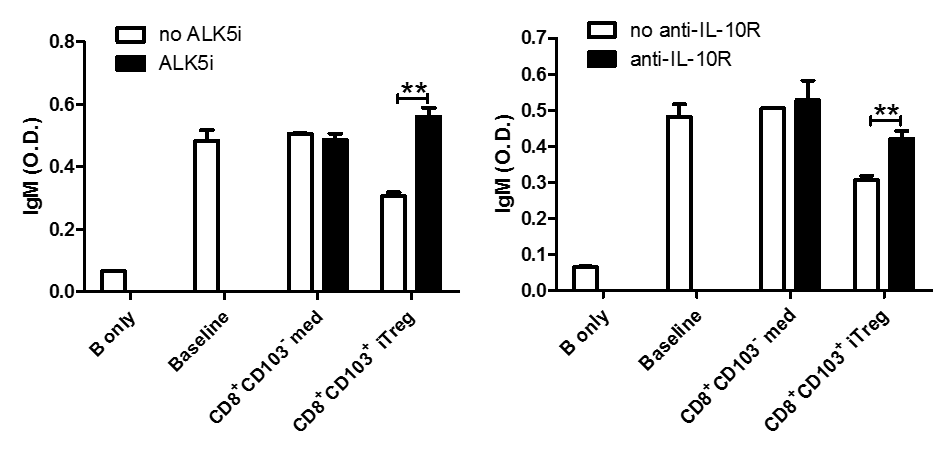
**

**Figure S2 | CD8^+^CD103^+^ iTregs suppress B cell responses *ex vivo* mainly by TGF-β or/and IL-10 signals.** Fresh B cells were co-cultured in 24 well plates with CD8**^+^**CD103**^-^** med or CD8^+^CD103^+^ iTreg (the ratio of T cells: B cells was 1: 2) in the presence of LPS, with or without ALK5i (DMSO) or anti-IL-10R (cIgG). The supernatants were collected 3 days after the co-cultures of T and B cells systems, and the IgM secretion was detected by an ELISA. Summarized data indicate the mean ± SEM of three independent experiments (***P<*0.01).

**Figure S3**


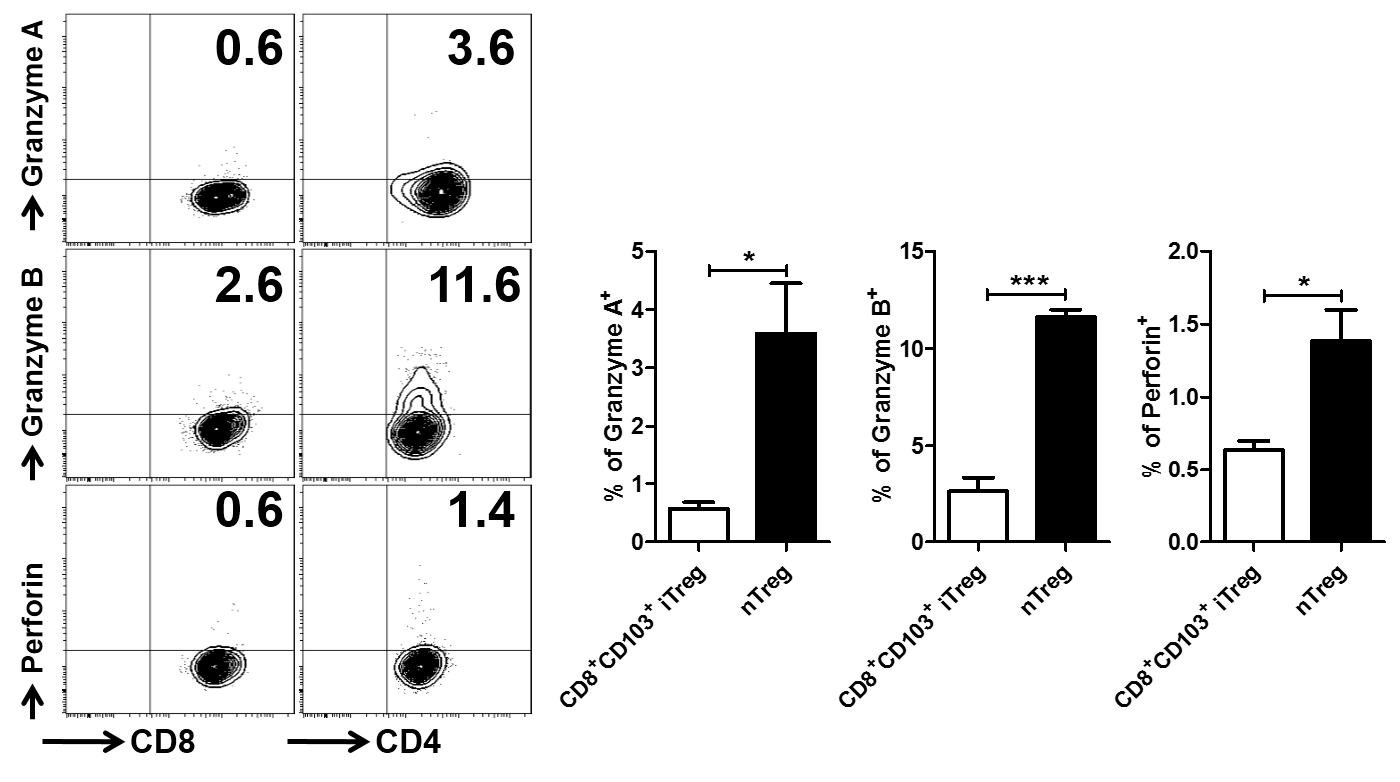


**Figure S3 | The expression of Granzyme A, Granzyme B or Perforin in CD8^+^CD103^+^ iTreg was less than nTreg.** CD8^+^CD103^+^ iTreg cells or nTreg were respectively stained with FITC- Granzyme A, Granzyme B, Perforin, then tested the expression of this three cytokines gated on CD8^+^CD103^+^ or CD4^+^CD25^+^ by ﬂow cytometry. The data indicate the mean ± SEM of three independent experiments. (**P<*0.05, ****P<*0.001, CD8^+^CD103^+^ iTreg *versus* nTreg).

**Figure S4**


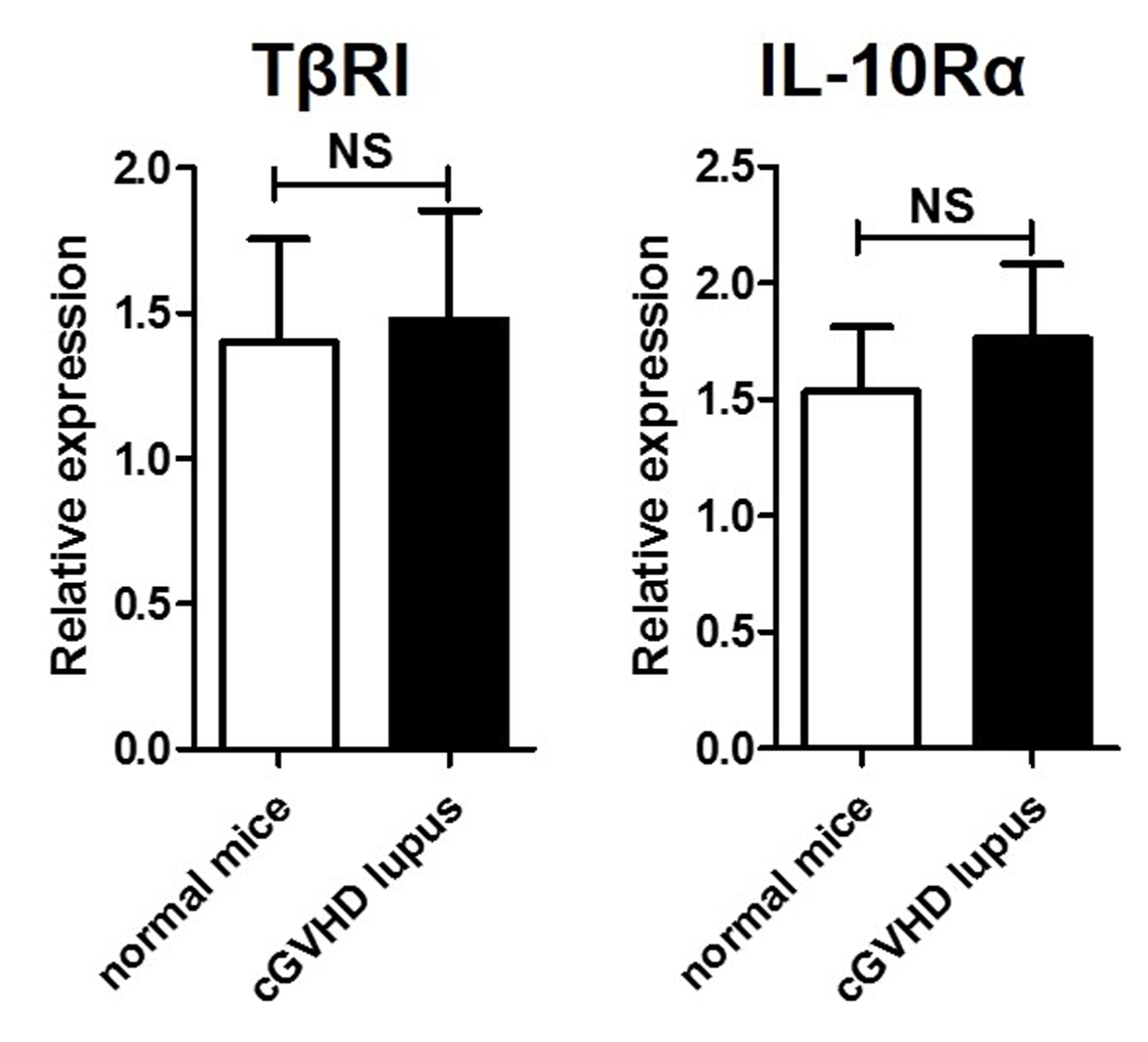


**Figure S4 | Expression of TβRI and IL-10Rα on cGVHD lupus mice and normal control.** Total RNA was extracted from B cells of cGVHD lupus mice or normal control mice. Real-Time PCR experiments were carried out. The data indicate the mean ± SEM of three independent experiments (NS means no significance, B cells from cGVHD lupus mice *versus* B cells from normal control mice).

**Figure S5**


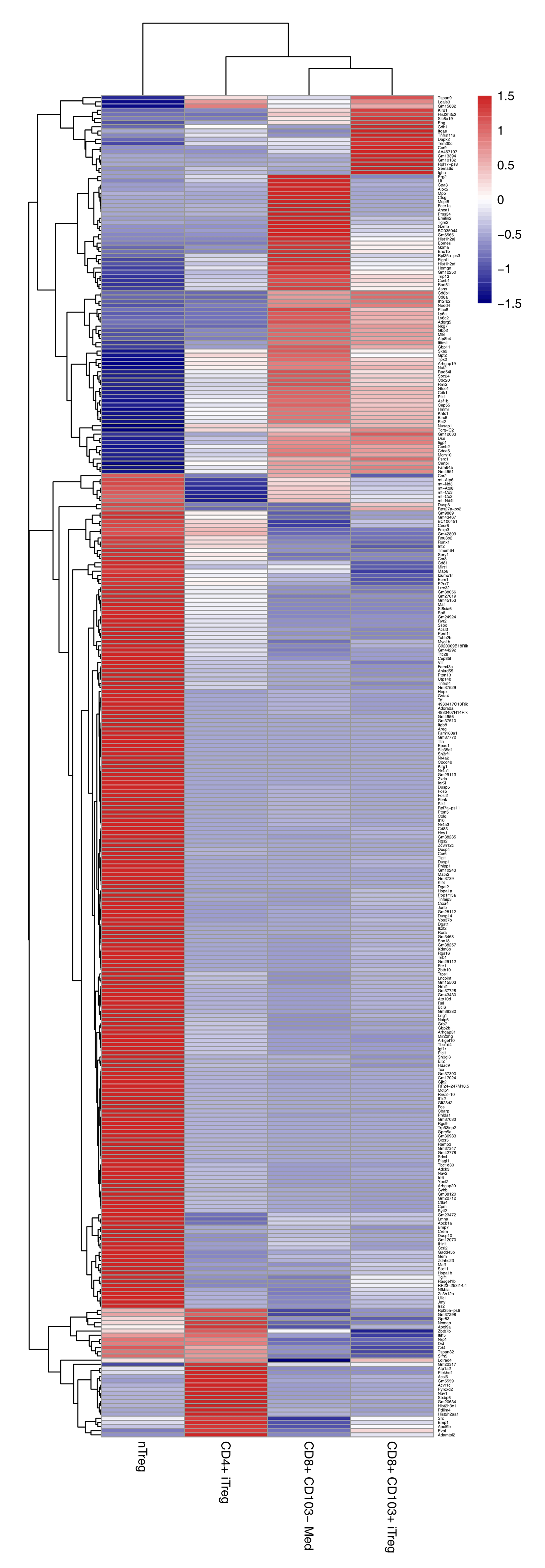


**A**

**
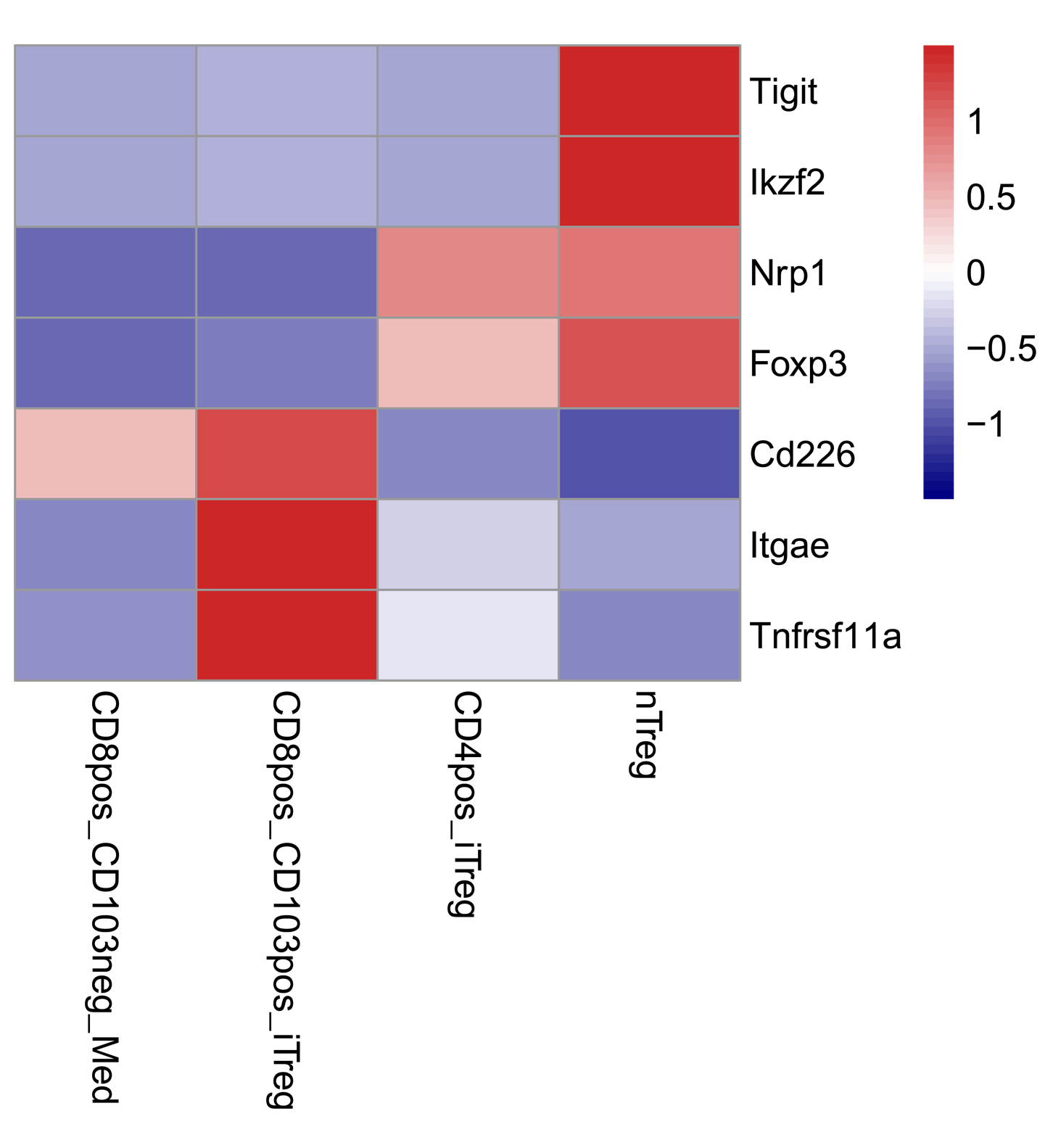
**

**B**

**Figure S5 | CD8^+^CD103^+^ iTreg, CD4^+^ iTreg and nTreg all have its own genes expression profiles.** RNAseq were conducted on an Illumina HiSeq platform following the manufacturer’s instructions. (**A**) The genes expression heatmap of different Treg types were shown. Each cell subset has its own specific gene profile that may be used as a tool to distinguish one population from another. (**B**) Potential cell markers for CD8^+^CD103^+^ iTreg, CD4^+^ iTreg and nTreg subsets

**Figure S6**


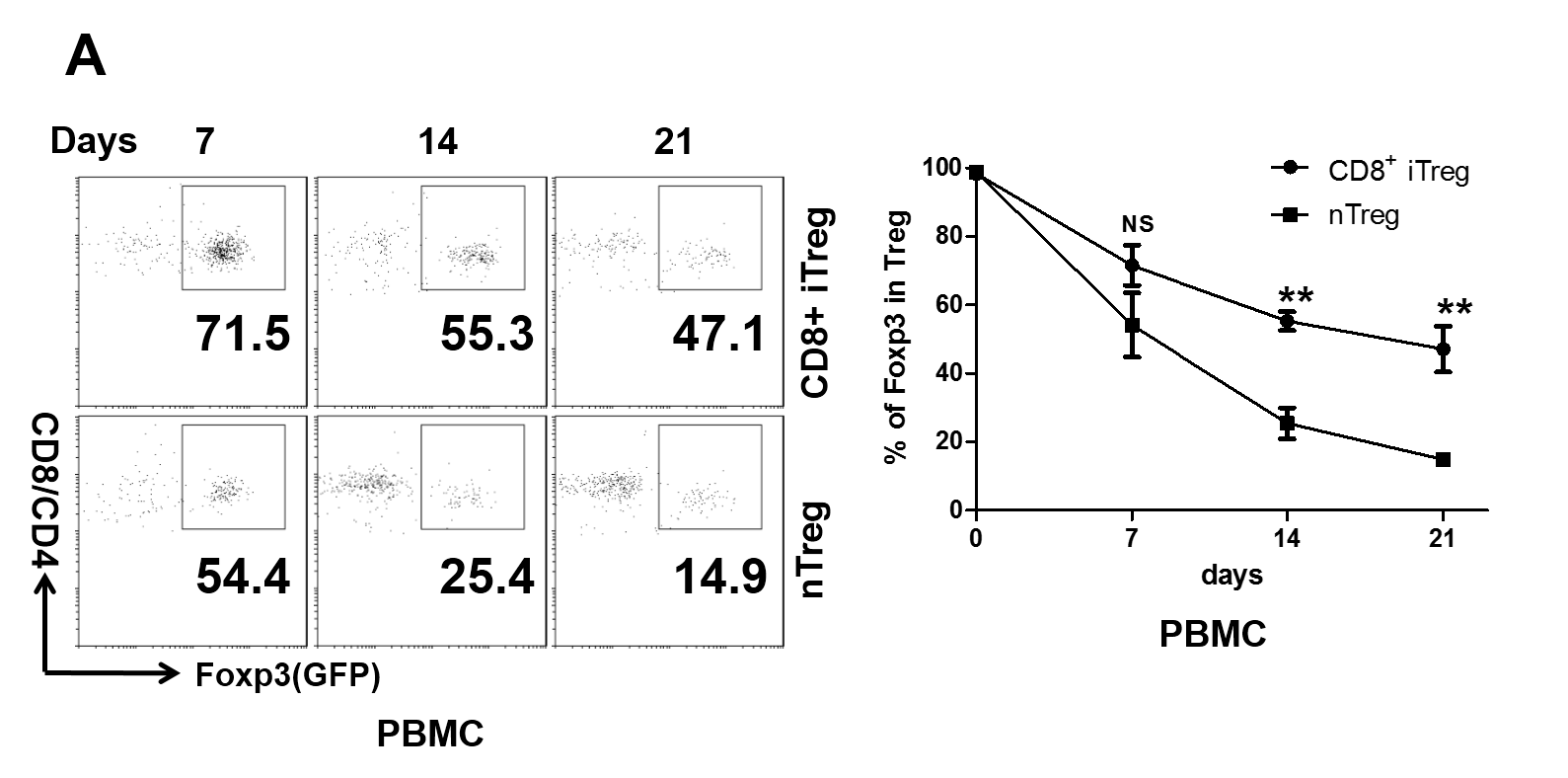


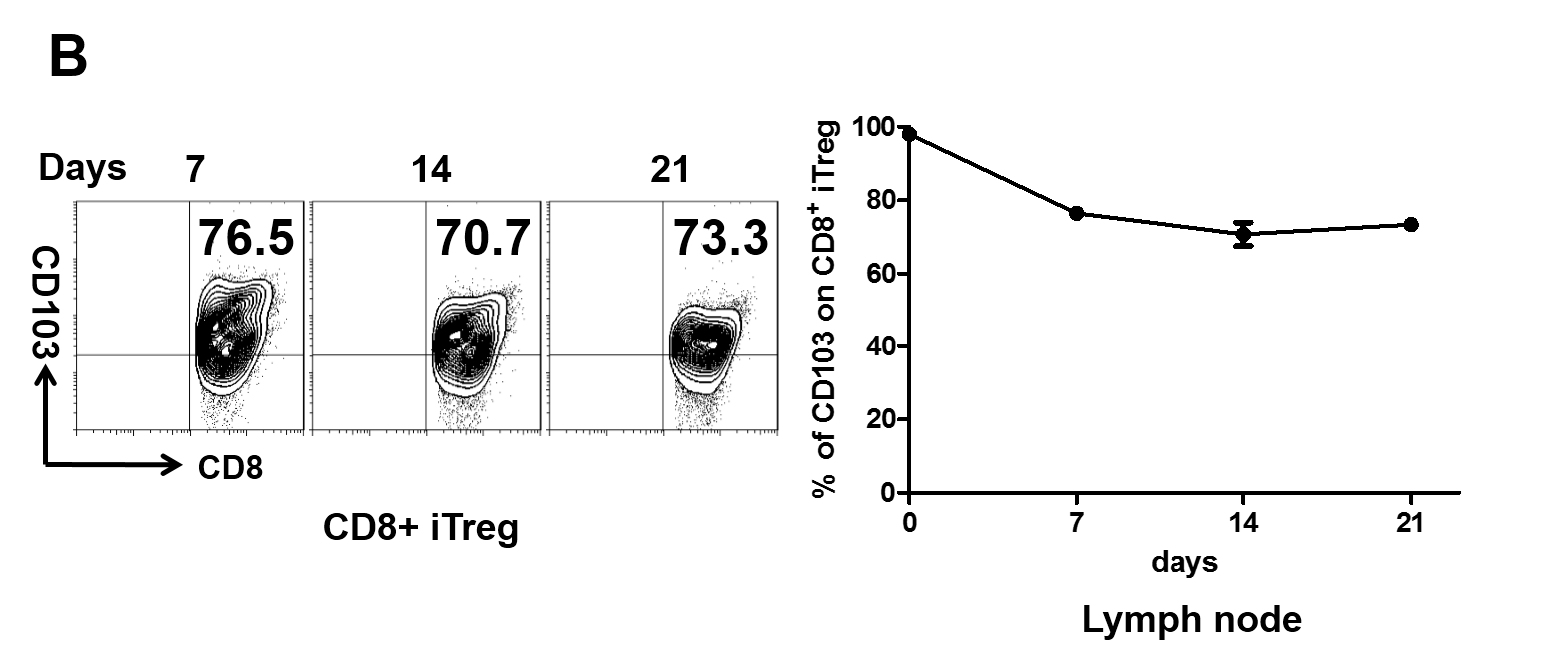


**Figure S6 | CD103 expression in CD8^+^CD103^+^ iTregs is more stable than the Foxp3 expression in nTreg *in vivo*.** Transferred purified CD8^+^CD103^+^Foxp3^+^ iTregs or CD4^+^Foxp3^+^ nTregs population into RAG1^-/-^ mice respectively and tested the CD103 or Foxp3 expression by ﬂow cytometry in days 7, 14 and 21. There were three mice in each group and experiments were repeated with similar results with three times. (A) The Foxp3 expression in CD8^+^CD103^+^ iTregs is more stable than that of nTreg in different time-points with significance. (B) CD8^+^CD103^+^ iTregs have stable and retentively high CD103 expression in different time-points *in vivo*. The data indicate the mean ± SEM of three individuals (NS means no significance, ***P<*0.01, CD8^+^ iTreg *versus* nTreg).
